# Campylobacter fetus subsp. fetus: an unforeseen cause of abortion in regional Australia

**DOI:** 10.1099/acmi.0.000889.v5

**Published:** 2025-02-14

**Authors:** Immanuella Owusu-Ansa, Manjeera Ramadas, Nikhitha Jacob, Femi E. Ayeni

**Affiliations:** 1 Central Gippsland Health, Melbourne, VIC 3850, Australia; 2 Department of Surgery, Nepean Hospital, Kingwood, NSW 2747, Australia; 3 Nepean Clinical School, The University of Sydney, Sydney, NSW 2747, Australia

**Keywords:** abortion, *Campylobacter fetus*, Gram-negative bacterium, infectious disease, pyrexia of unknown origin, unpasteurized milk

## Abstract

*Campylobacter fetus (C. fetus)* subsp. *fetus,* a Gram-negative bacterium, is an established cause of abortion and infertility in cattle, sheep and goats. Human infections have been rarely reported. In contrast to TORCH infections, this *Campylobacter* species is hardly recognized as a cause of abortion in humans. Since 1947 after the first case report in France, there have been only 11 reported cases of pregnant women worldwide and no published reports in Australia, pregnant or otherwise. The case in this study was compared to the first reported infection of *C. fetus* in 1947 to raise awareness and educate doctors and midwives, subsequently impacting prenatal and antenatal counselling in these regions. A Venn diagram was constructed to highlight the similarities between this and the index case. The similarities found included the clinical state of the patient post-abortion and the all-important history of exposure to farm animals that suffered recent deaths on the farms of both patients. Some of the differences included the time of onset of symptoms to the time of abortion, the choice of antibiotics by both treating teams and the presentation of sepsis, suggesting the importance of *C. fetus* subsp. *fetus* as a perinatal infection.

## Data Summary

All data needed for review are presented in the report.

## Introduction

*Campylobacter fetus*, a Gram-negative bacterium, is one of the most under-recognized species within the genus *Campylobacter*. This species has two subtypes: *C. fetus* subsp. *fetus* and *C. fetus* subsp. *venerealis* [1]. *C. fetus* has long been acknowledged as an infectious agent of animals involved with spontaneous abortion in cattle and sheep, predominantly *C. fetus* subsp. fetus [2]. The primary reservoir of *C. fetus* subsp. *fetus* is the gastrointestinal tract of sheep and cattle [3]. This subsp. has also been isolated in the faeces of other animals [1]. Interestingly, the primary reservoir of *C. fetus* subsp. *venerealis* is the bovine genital tract, causing predominantly infectious infertility and abortion [3]. While being a common cause of abortion in bovines, *C. fetus* does not commonly cause septic abortion in humans [3]. Only several cases of *C. fetus*-associated abortions have been reported worldwide, with no cases reported in Australia [1]. This case study explores the first reported case of abortion induced by *C. fetus* subsp. *fetus* in Australia. This report presents a comparative study between the current case and the first documented *C. fetus* infection in 1947. Furthermore, the importance of *C. fetus* as a cause of febrile illness in pregnancy in regional Australia, especially in farming communities has been outlined.

## Methodology

### Case presentation

A 28-year-old female, G1P0, 18 weeks pregnant, presented to a rural emergency department with a high-grade fever that began the previous night. She reported no other symptoms. Initial assessment revealed that the patient had a temperature of 39.7 °C, a pulse rate of 140 bpm and a blood pressure of 14/9 kPa. Obstetric examination, including bedside foetal ultrasound, revealed live intra-uterine pregnancy. Suspecting maternal sepsis, clinicians initiated a comprehensive septic workup and promptly commenced ceftriaxone for presumed pyrexia of unknown origin (PUO). Blood cultures and other blood investigations were obtained during admission (Tables1  2). Seven hours post-admission, the patient experienced a sudden onset of severe back pain, resulting in an unfortunate spontaneous abortion of the foetus by midday, with some similarities to the first documented *C. fetus* infection reported in 1947. After the miscarriage, placental histopathological examination, microbiological examination of the placenta and foetus and chromosome analysis were done postmortem, where placental and foetal swabs were collected (Table 3).

**Table 1.** Trend of white cell count (WCC), neutrophils and C-reactive protein (CRP) during admission

	WCC (×10^9^ l^−1^)	Neutrophil (×10^9^ l^−1^)	CRP (mg l^−1^)
Day 1	27.8	25.3	54
Day 2	29.3	26.7	167
Day 3	9.6	7.4	78

**Table 2.** All blood investigation results

Blood test	Result
Blood group	A+
Urine MCS	NAD
Blood culture	Positive for *C. fetus*
Rubella	Immune
Hepatitis B and C	Negative
Varicella-zoster	Immune
Vitamin D	NAD
Viral PCR	Negative
HIV	Negative

MCSmicroscopy, culture, and sensitivityNADno abnormality detected

**Table 3.** Results of placental and foetal swabs

Location	Result
Foetal: left and right groin	Culture: light growth of *C. fetus,* no Group B strep, no mycoplasma hominis
Foetal: left and right Axilla	Culture: light growth of *C. fetus,* no Group B strep, no mycoplasma hominis
Foetal: mouth	Moderate growth of *C. fetus,* light growth of skin flora
Placental: maternal	Culture: moderate growth of *C. fetus*
Placental: foetal	Culture: Light growth of *C. fetus*

### Treatment

Resuscitation with 2 l of sodium chloride was commenced immediately. Additionally, a stat dose of 2 g intravenous ceftriaxone was commenced, and paracetamol was given. Unfortunately, this did not halt the progression of the febrile illness, subsequently leading to the abortion of the foetus. After *the C. fetus* was isolated, the patient was prescribed 500 mg of oral azithromycin daily for 1 week (as per the advice from Infectious Disease). More importantly, she was provided with grief counselling and psychosocial support.

### Results

Laboratory findings during admission demonstrated elevated neutrophils and a CRP of 167 mg l^−1^, both of which normalized rapidly following the miscarriage (Table 1). Blood cultures obtained during admission yielded a positive result for *C. fetus*. Placental histopathology revealed evidence of chorionic plate vasculitis that appeared secondary to the dense chorionic neutrophil infiltration. Additionally, perivillous fibrin deposition increased globally, and there was suppurative neutrophilic intervillositis with chorionic villous necrosis and destruction. The membranes also had similar results of dense neutrophilic infiltration and decidual necrosis. A thrombus was identified peripherally in the placenta with adjacent inflamed and necrotic decidua, which might suggest an indication that *C. fetus* infection caused the abortion. Additional serology/investigations were unremarkable, as shown in Table 2. The microscopy revealed no bacteria; however, the culture revealed the growth of *C. fetus* (Table 3). Placental and foetal swabs revealed growth of *C. fetus* (Table 3). No chromosomal defects were identified, and no abnormalities were noted with the umbilical cord except for the thrombus.

## Discussion

The genus *Campylobacte*r consists of 26 species and 9 subsp.; however, the taxonomy of the genus is constantly changing due to reclassification and the discovery of novel species [4]. As an example, *Campylobacter butzleri* was previously renamed *Helicobacter butzleri*. A few in other taxa are now also classified as *Campylobacter*; *Bacteroides ureolyticus* is now classified as *Campylobacter ureolyticus* [5]. Currently, this species has two subtypes of *C. fetus: C. fetus* subsp. *fetus* and *C. fetus* subsp. *venerealis. C. venerealis* has been isolated in the genital part of cows and has been named a culprit of infertility and sporadic abortions. Septic abortion, usually epizootic in sheep, has been historically associated with *C. fetus* subsp. fetus and, to a lesser extent, with *Campylobacter jejuni* [6].

**Fig. 1.**
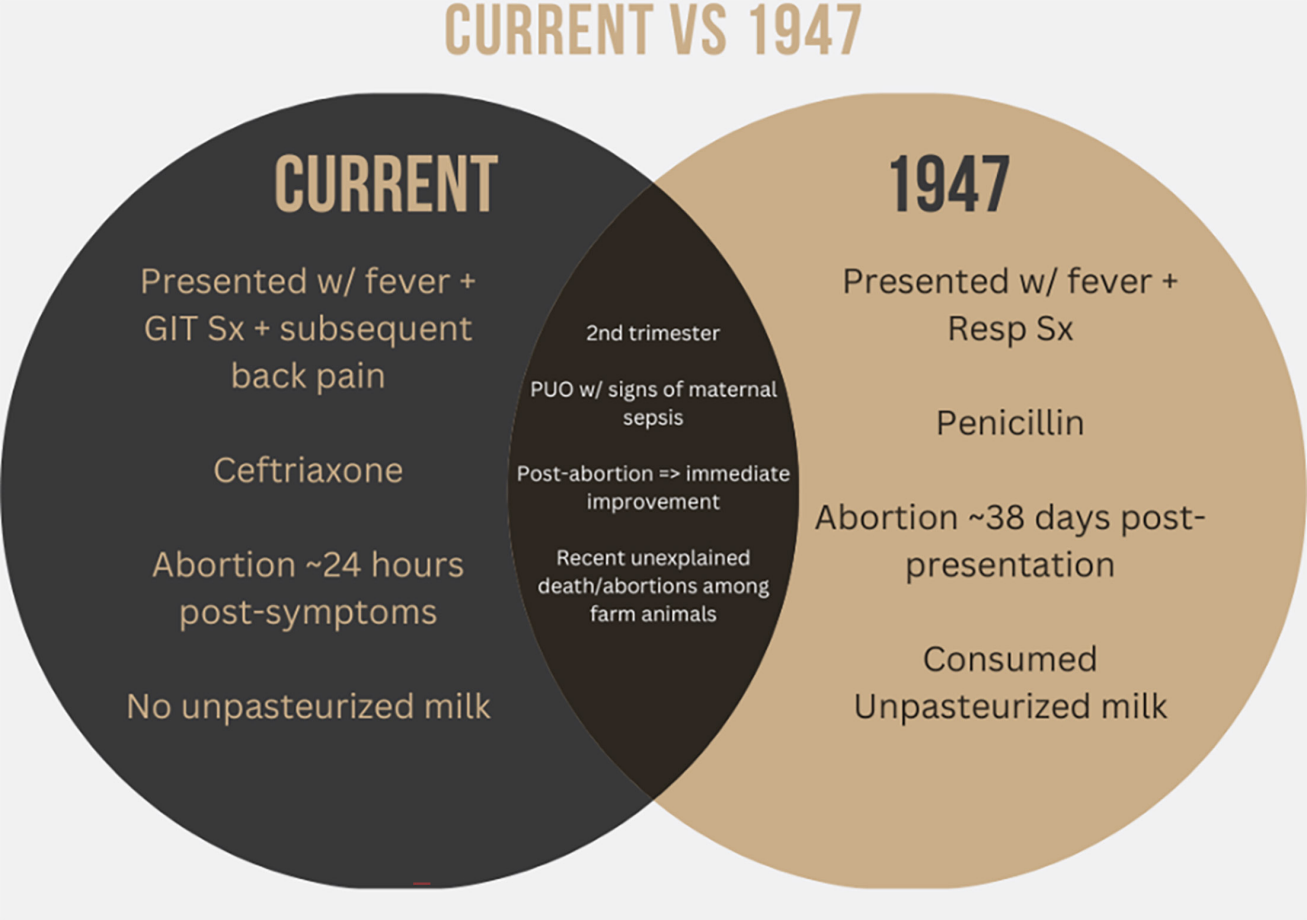
Similarities and differences shown in the Venn diagram between the current and 1947 case studies.

In 1957, a systematic study revealed that *Vibrio fetus* (now termed *C. fetus*) caused systemic illness, while the more common *C. Jejuni* and *Coli* caused diarrheal illnesses [7]. Interestingly, *C. fetus* exclusively is known to cause bacteraemia. This bacterium is known to be zoonotic in origin. It primarily resides in the gastrointestinal tract of sheep and cattle, with * C. fetus* subsp. *fetus* being the primary reservoir. However, it can also be found in the faeces of other animals. The very first case of abortion in humans due to a *C. fetus* was reported in 1947 by Vincent, Dumas and Picard [8]. This case involved a 39-year-old woman, Para 6, with no significant co-morbidities, who presented to a physician with fever, syncope, chills and coughing. Her examination revealed basal crepitations in the lung base. She was treated with penicillin for 36 days without improvement. Unfortunately, she aborted 36 days after the onset of symptoms. Interestingly, it was reported that the patient had consumed unpasteurized milk from a farm with reported cases of abortions in animals, an apparent similarity with this case (Figs1  2).

**Fig. 2.**
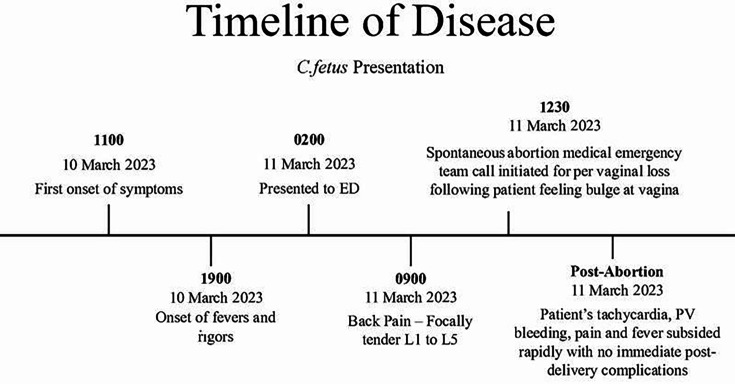
Timeline of disease events from onset of symptoms to abortion.

In contrast, the initial history revealed no potential exposures to perinatal infections (unpasteurized milk consumption); however, a more comprehensive evaluation following the foetal demise identified close contact with farm animals and a history of recent bovine abortions on her husband’s farm. Interestingly, following the abortion, the patient’s symptoms also abated spontaneously (Fig. 2). This intriguing observation suggests a possible association between bacterial presence and symptom resolution, which merits further investigation to elucidate the underlying pathogenesis.

As depicted in the Venn diagram in Fig. 1, in the case reported [8] from France, the patient presented in the second trimester with overwhelming signs of sepsis; this was seen in the current case report from rural Australia. Additionally, there was complete resolution of the symptoms of infection as soon as the foetus was terminated, as seen in this case report timeline above in Fig. 2; the same was reported in the French case. One may even be bold enough to conclude that the bacteria may have an affinity for the placenta regarding its pathogenesis.

In 1947, the patient reported predominantly respiratory sepsis as opposed to PUO with severe abdominal pain in this case. Although there was a resolution of symptoms post-abortion, it only took 24 h for the patient’s termination to occur, while it took 38 days from the time of infection to the abortion of the foetus in the 1947 case. This difference raises even more questions: What delayed the disease’s progression? Could it be the antibiotic choice? Ultimately, penicillin was used, while ceftriaxone was used in the current case to treat the infection.

Compared to *C. Jejuni* and *Coli*, little is known about the disease progression of *C. fetus*. Accordingly, in farming communities in Australia, rural doctors and midwives need to be aware of this bacterium and raise awareness through education and proper history-taking. Simply asking patients about the presence of farm animals and reporting animal deaths, including perinatal deaths, is key. The alarming lack of awareness regarding the ability of this bacterium to infect both humans and animals poses a significant threat to pregnant women residing in these areas. There is much room for research and probing into the pathogenesis and disease progression of *C. fetus* subsp. *fetus*. There is also potential for pharmacological research into the choice of drugs for treatment if the disease is suspected.

Moreover, there is room for further research to consolidate the transmission mode; the potential of enteral contact and even sexual transmission has been proposed in animals; however, it has not been proven in humans [1,9,13].

## Conclusion

This research revealed *C. fetus* subsp. *fetus* in both the mother and the foetus. This bacterium is well established as a cause of abortion in farm animals such as cattle and sheep but not well established in humans. This study compared the first reported case in the world, a rural area in France in 1947 to create awareness among health workers, especially those in rural farming areas in Australia, and to educate them about *C. fetus* infection. This case may also suggest a couple of research objectives in microbiology and infectious disease. Though efforts to prevent the patient from losing her pregnancy were not successful, this case opens a box of unanswered questions about *C. fetus* subsp. *fetus* and its importance as a perinatal infection. Perhaps in the future, it may earn a spot in the well-known acronym for TORCH infections.

## Human and animal rights

All the procedures performed in this study followed the ethical standards of the 1964 Helsinki declarations. This report does not contain any animal studies performed by any of the authors.

## Abbreviations

CRP: C-reactive protein
PUO: pyrexia of unknown origin
WCC: white cell count
